# Retention of Placenta

**Published:** 1882-10-20

**Authors:** J. V. White

**Affiliations:** Au Sable, Michigan


### RETENTION OF PLACENTA.

BY J. V. WHITE, M.D., C.M., M.C.P. & S., ONT., AU SABLE, MICHIGAN.

The expediency of removing the retained placenta under certain circumstances is worthy of the most careful attention, though how often do we notice the bad effects arising from a premature action taken by the physician in order to meet previous engagements. One of the important rules of long standing, that the placenta, if retained, should always be removed, is now, almost without exception, so invariably followed that its greatness can scarcely be fully realized. Certain conditions do arise, from time to time, when it is very difficult, and in some instances impossible, to be governed by this rule entirely; and some cases occur in which there has been a difficulty in recognizing the fact that either a portion has remained attached, or some abnormal contraction of the uterine organ has been the cause of retention.

The firm closure of the uterus, and the firmness of its adhesions, are among the predominating causes that prevent its entire removal. Whenever we have to deal with premature expulsion of fœtus, then our hopes of introducing the hand is generally retarded. Seldom it is in the fully developed organ that we are unable, at any rate with the assistance of chloroform, to pass our hand sufficiently far to empty the uterus. Besides these obstacles the condition of our patient must be considered before we attempt its removal; as, for instance, through the already arrested hemorrhage; although, should our patient be the victim of severe flooding, it would be highly imprudent to wait, because the shock of operating would be less injurious to the system than the depletion; whereas, if the hemorrhage has been checked, beside saving patient from shock of manipulating, it will be an advantage to wait till the shock of bleeding has passed and circulation established from the smaller vessels, and the heart restored to its proper tone.

Before I leave the subject I wish to draw attention to another point, viz.: œdema of vulva most frequently following primipara cases, and consider it a very annoying impediment, especially when the case is of long standing and the parts so sensitive to the touch from the vaginal secretions that continually pass. To illustrate the ideas:

I was called to see Mrs. —— on May 14th; found her suffering from severe pain in back and extending to her maternal parts. I ordered Dover's powder and rest in recumbent position, and before she attempted to walk to put on her abdominal support. This being done, she received relief in a few days. About the 1st of July she went to visit her relations. However, she was not there over a week until her husband wrote me, stating his wife's feet and eyes were swollen badly. I ordered acet. pot., bicarb. pot. and tr. digitalis, with an alkaline laxative. She improved rapidly, and on the 13th was delivered of a child (very small), before I could get there. She was attended by a physician who was desirous of attending the case, and to complete it before I could arrive. He made extra exertion toward removing the placenta, made traction on the cord, and pressure on fundus, all to no avail. Gave ergot F. E. and used friction, all to no appreciable benefit. When I arrived I found the vulva very tender and swollen, and the patient would cry and turn all colors at any attempt of handling the cord; but, through my persistence, I made an examination and found a patent os sufficient to admit one finger. I deemed it necessary to give her rest, and administered pot. brom. grs. 44, so she rested for six hours quietly. By this time tenderness had almost disappeared from vulva, and could stand any traction I found necessary. Made another examination, and found os the same; then, determined to complete the case, gave chloroform. I then made traction once more, but no advancement; so I introduced my hand well up into the uterus and explored it, discovering a hard attached mass well up on the right side. With my fingers I detached the adherent portion with some difficulty, followed by a severe hemorrhage. Ordered ergot F. E. ʒ ss. and, retaining hand in uterus, made pressure on fundus with the other. Shortly I had contraction, and elevated the foot of the bed to extent of six inches, nothing remaining in half an hour but slight bloody mucus discharge. Ordered carbolic acid injection, 1 in 80, and she made a rapid recovery, making 24 hours from birth of child to the time the placenta was taken.

From a previous examination of her urine, slight traces of albumen were found, which might be indications of being affected with albuminaria during the last months of pregnancy. However, the symptoms were somewhat indicative, since swelling of eyelids (lower) and œdema of legs and vulva, and yielding to the alkaline treatment. The pain that was complained of during my attendance, with sensation of heat about the eighth month, I now consider the results of inflammatory action going on in the placenta: the scrotinal surface was yellow and thickened; and the part firm and consolidated to the extent of about an inch.

This case is one of the few that lead to such rapid recovery.—*Detroit Clinic.*

